# Rapid induction of transdermal buprenorphine to subcutaneous extended-release buprenorphine for the treatment of opioid use disorder

**DOI:** 10.1186/s13722-024-00479-1

**Published:** 2024-06-18

**Authors:** Pouya Azar, Hannah Schneiderman, Henry Barron, James S. H. Wong, Maximilian Meyer, Dayyon Newman-Azar, Matin Narimani, Martha J. Ignaszewski, Nickie Mathew, Rodney Mullen, Reinhard M. Krausz, Anil R. Maharaj

**Affiliations:** 1 Integrated Psychiatry, Pain, and Addiction Service, Vancouver General Hospital, Flr 8-2775 Laurel St, V5Z 1M9 Vancouver, BC Canada; 2 Department of Psychiatry, Faculty of Medicine, University of British Columbia, Vancouver, BC Canada; 3 School of Medicine, University College Cork, Cork, Ireland; 4 Addictions and Concurrent Disorders Research Group, Department of Psychiatry, Faculty of Medicine, University of British Columbia, Vancouver, BC Canada; 5 Clinic of Adult Psychiatry, University of Basel Psychiatric Clinics, University of Basel, Basel, Switzerland; 6 School of Biomedical Engineering, Faculty of Medicine, Faculty of Engineering, University of British Columbia, Vancouver, BC Canada; 7 Substance Use Response and Facilitation Service, BC Children’s Hospital, Provincial Health Services Authority, British Columbia, Canada; 8 BC Mental Health & Substance Use Services, Provincial Health Services Authority, British, Columbia Canada; 9 The C4 Foundation, Coronado, CA USA; 10 Pharmacokinetics Modeling and Simulation Laboratory, Faculty of Pharmaceutical Sciences, University of British Columbia, Vancouver, BC Canada

**Keywords:** Low-dose induction, Micro-induction, Micro-dosing buprenorphine, Transdermal, Subcutaneous, Butrans, Sublocade

## Abstract

**Background:**

Buprenorphine is an effective and safe treatment for opioid use disorder, but the requirement for moderate opioid withdrawal symptoms to emerge prior to initiation is a significant treatment barrier.

**Case Presentation:**

We report on two cases of hospitalized patients with severe, active opioid use disorder, in which we initiated treatment with transdermal buprenorphine over 48 h, followed by the administration of a single dose of sublingual buprenorphine/naloxone and then extended-release subcutaneous buprenorphine. The patients did not experience precipitated withdrawal and only had mild withdrawal symptoms.

**Conclusions:**

This provides preliminary evidence for a rapid induction strategy that may improve tolerability, caregiver burden, and treatment retention as compared to previous induction strategies.

## Background

Buprenorphine is a partial µ-opioid receptor agonist widely used for the treatment of opioid use disorder (OUD) [1]. It has a superior safety profile compared to full µ-opioid receptor agonists due to its ceiling effect for respiratory depression [2]. However, its high binding affinity leads to displacement of other opioids during induction, which can cause precipitated withdrawal [3]. To avoid this phenomenon when being started on buprenorphine, patients are typically instructed to abstain from opioids until objective withdrawal symptoms emerge. This is a major treatment barrier, as many patients are unwilling to undergo this period of opioid withdrawal [4].

To date, several alternative induction strategies have been used to combat the challenges of standard induction. In the low-dose induction protocol (originally known as the Bernese method, and previously known as micro-dosing and micro-induction), low doses of buprenorphine are administered multiple times per day, combined with a full µ-opioid agonist, such as hydromorphone, removing the need for withdrawal symptoms to emerge prior to treatment initiation [5]. Our team has developed several accelerated low-dose induction protocols, built upon the Bernese method, the most recent of which involves the use of transdermal buprenorphine (BUP-TD), followed by a switch to sublingual buprenorphine (BUP/NX) in 48 h [6–10]. This method, known as the IPPAS method, involves the application of twelve 20 µg/h patches (BuTrans®) over 48 h, and provides similar predicted plasma concentrations achieved by our SL rapid low-dose protocol, which has been clinically robust at our hospital and used for hundreds of patients [6, 10, 11]. After a two-day induction period, the patient is switched to a therapeutic dose of SL buprenorphine/naloxone.

**Fig. 1:**
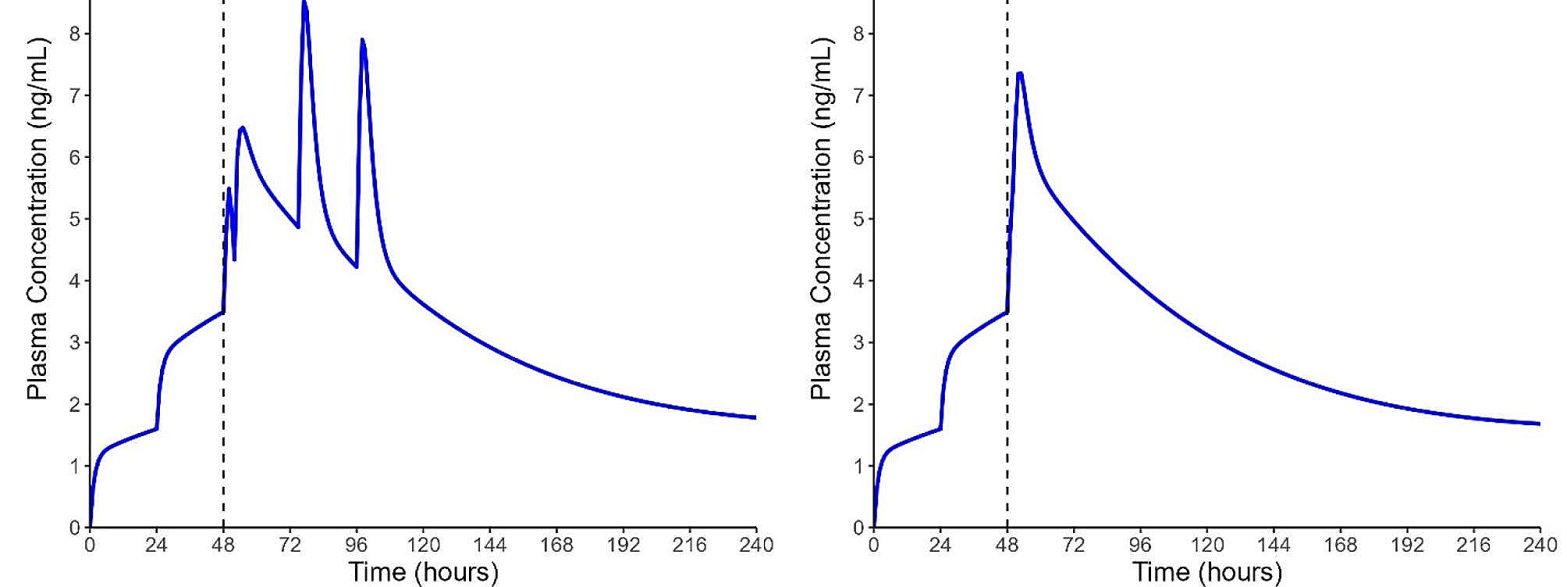
Pharmacokinetic model simulations of buprenorphine plasma concentration-time values for patients presented in Case 1 (A) and Case 2 (B). BUP-TD patches were applied at 0 and 24 h. At 48 h (dotted line), all applied patches were removed and BUP-XR was administered. In Case A, additional 4 mg doses of BUP/NX were administered for withdrawal management. Plasma concentration modelling was performed using an integrated model combining two previously published population pharmacokinetic models: Priestley et al. [13] (BUP-TD and BUP/NX) and Jones et al. [14] (BUP-XR). Sublingual bioavailability was reduced to 0.35 (from 0.426) in Priestley et al.’s model to account for the reduced bioavailability of sublingual tablets compared to the buccal film formulation. Pharmacokinetic analysis was performed in R v4.0.3 using the RxODE package [15]. Our BUP-TD protocol provides similar predicted plasma concentrations to our SL rapid low-dose induction protocol [10].

The IPPAS method is rapid, convenient, and reduces the chance of withdrawal symptoms, which may improve tolerability and treatment retention. In this paper, we sought to utilize the IPPAS method to start patients on injectable long-acting buprenorphine, Sublocade® (BUP-XR), following 48-hour rapid induction with BUP-TD. Compared to daily standard of care medication (liquid methadone or BUP-SL), BUP-XR has been shown to provide longer treatment retention, greater opioid abstinence, and less opioid craving [12]. In this paper, we describe two cases of patients who were successfully started on buprenorphine using this method. Pharmacokinetic predictions of buprenorphine plasma concentration with time were generated for each case (Fig. 1).

## Case Presentation

### Case 1

A 52-year-old man on disability, residing in supported housing, was admitted to a tertiary care hospital for acute kidney failure. His past medical history included hepatitis C, laparoscopic cholecystectomy for gallstones, left knee injury, and a previous nasal fracture. His substance use history included stimulant and opioid use disorders for 10 years, starting with daily cocaine and intermittent heroin use. Two years prior, he had transitioned to using unregulated fentanyl. Prior to admission, he was using 200 mg of intravenous and inhaled unregulated fentanyl per day. He had a past trial of methadone up to 80 mg daily, but discontinued treatment as he felt fentanyl helped him balance out the effects of stimulants. He had also been offered SL BUP/NX, but the fear of withdrawal symptoms during the induction period had discouraged him from taking it. On the day of admission, he reported that he last used cocaine the same morning and last used fentanyl the day prior. His urine drug screen tested positive for fentanyl, methadone, opiates, and cocaine.

On the day of admission, he received 8–16 mg oral (PO) hydromorphone every 1 h (q1h) as needed for the management of pain and opioid withdrawal. He expressed that his goal was abstinence from unregulated opioids, noting that lately he was using opioids primarily to prevent withdrawal symptoms. Furthermore, he was interested in the BUP-XR depot because he found daily medication administration challenging.

He subsequently completed a BUP-TD induction over 48 h while continuing to receive hydromorphone as needed (Table 1). On the first day of induction, six 20 µg/h TD buprenorphine patches were applied to the patient’s back. After 24 h, six additional patches were applied. All patches were removed 48 h post induction, and he received 4 mg SL BUP/NX. He then received 300 mg SC BUP-XR four hours later. No precipitated withdrawal occurred during the induction period, as the clinical opiate withdrawal scale (COWS) score at baseline was 6 and subsequently decreased [16]. He received 4 mg SL BUP/NX PRN for withdrawal management, which was discontinued upon discharge. He was provided with an appointment with an outpatient addiction psychiatrist to receive his next BUP-XR injection. He remained on BUP-XR for several months prior to relapse. In his follow-up appointments preceding relapse, there were no reports of withdrawal symptoms or overdoses.

**Table 1:** Transdermal Buprenorphine to Buprenorphine Extended-Release Induction Titration Schedule of Case 1

	Buprenorphine (Transdermal Patch)		Buprenorphine/Naloxone* (Sublingual Tablet)		BUP-XR (Subcutaneous Injection)	Hydromorphone			Clinical Opiate Withdrawal Scale (COWS)
	Dosing	Total dose	Dosing	Total dose	Dose administered	Dosing	Total dose	Oral Morphine Equivalents	
Admission day, preinduction						8–16 mg PO q1h PRN	24 mg	96 mg	
Preinduction day						8–16 mg PO q1h PRN	36 mg	144 mg	
Preinduction day						8–16 mg PO q1h PRN	44 mg	176 mg	
Preinduction day						8–16 mg PO q1h PRN	52 mg	208 mg	
Induction Day 1	6 × 20 mcg/h	120mcg				8–16 mg PO q1h PRN	44 mg	176 mg	Mean: 4.4 Range: 4–6
Induction Day 2	6 × 20 mcg/h	120mcg				8–16 mg PO q1h PRN	36 mg	144 mg	Mean: 2 Range: 0–4
Postinduction stabilization	Discontinued		4 mg OD	4 mg	300 mg SC	Discontinued		Discontinued	Mean: 0 Range: 0
Postinduction stabilization			4 mg OD	4 mg					Mean: 0 Range: 0
Postinduction stabilization, discharge day			4 mg OD and discontinued upon discharge	4 mg					Mean: 4 Range: 4

*Expressed as mg of buprenorphine component

*BUP-XR = buprenorphine extended-release injection*

*Q__ H = every __ hours*

*OD = once a day*

*PRN = as needed*

*PO = by mouth*

*SC = subcutaneous*

### Case 2

A 34-year-old woman, receiving social support for disability, and living with her mother in a private residence, was admitted to the same tertiary care hospital for asthma exacerbation and pneumonia. Her medical history included asthma, brachial plexopathy, and a right-hand fracture in 2018. Psychiatric history was significant for major depressive disorder (treated with sertraline), and self-reported ADHD. Her substance use history included opioid and stimulant use disorders, beginning four years prior. On admission, she endorsed smoking approximately 1 gram of unregulated fentanyl. She denied intravenous drug use or history of overdose. Past opioid agonist treatment (OAT) included BUP-XR depot injections (Sublocade®), which she was no longer taking. More recently, she was started on 500 mg daily of slow-release oral morphine (SROM, Kadian®), but had ongoing fentanyl due to cravings and withdrawal symptoms. She reported that her last fentanyl use was on the day of admission. Her urine drug screen was positive for fentanyl and opiates, and negative for cocaine, benzodiazepines, cannabis, and amphetamines.

On the day of admission, she was treated with azithromycin and ceftriaxone by the internal medicine team for pneumonia. She received 500 mg of SROM once daily, as well as the following medications for withdrawal management: 10-20 mg of oral morphine every 4 h as needed, 8-32 mg of oral hydromorphone every 3 h as needed, and 4-16 mg of subcutaneous hydromorphone every 3 h as needed. She reported a goal of abstinence from unregulated opioids, and subsequently underwent a BUP-TD induction over 48 h onto SC BUP-XR (Table 2). On the first day of induction, six 20 µg/h BUP-TD patches were applied on the patient’s back. After 24 h, six more patches were applied. After 48 h, all twelve patches were removed, and the patient received 4 mg SL BUP/NX. After 50.5 h, she received a 300 mg SC injection of BUP-XR. No precipitated withdrawal occurred during the induction period, as indicated by her low scores on the Clinical Opiate Withdrawal Scale (COWS), ranging from 1 to 5, i.e., only mild objective withdrawal symptoms. The patient noted that she tolerated the induction well and denied any side effects. A follow-up appointment was scheduled with her general practitioner for her next BUP-XR injection. She remained on BUP-XR for several months before relapse. In her follow-up appointments prior to relapse, there were no reports of withdrawal symptoms or overdose.

**Table 2:** Transdermal Buprenorphine to Buprenorphine Extended-Release Induction Titration Schedule of Case 2

	Buprenorphine (Transdermal Patch)		Buprenorphine/Naloxone* (Sublingual Tablet)		BUP-XR (Subcutaneous Injection)	SROM/Morphine/HM			Clinical Opiate Withdrawal Scale (COWS)
	Dosing	Total dose	Dosing	Total dose	Dose administered	Dosing	Total dose	Oral Morphine Equivalents	
Induction Day 1	6 × 20 mcg/h	120mcg				SROM 500 mg PO OD Morphine 10 mg PO Q4H PRN HM 8-32 mg PO q3H OR 4-16 mg SC q3H PRN	SROM 500 mg Morphine 10 mg	510 mg	Mean: 1.33 Range: 1–3
Induction Day 2	6 × 20 mcg/h	120mcg				SROM 500 mg PO OD HM 8-32 mg PO q3H OR 4-16 mg SC q3H PRN	SROM 500 mg	500 mg	Mean: 3 Range: 1–5
Postinduction, discharge day	Discontinued		4 mg OD PRN	4 mg	300 mg SC	Discontinued		Discontinued	Mean: 1 Range: 1

*Expressed as mg of buprenorphine component

*BUP-XR = buprenorphine extended-release*

*q __ h = every __ hours*

*OD = once a day*

*PRN = as needed*

*PO = by mouth*

*SC = subcutaneous*

*SROM = slow-release oral morphine*

*HM = hydromorphone*

## Discussion

This case series presents two patients with severe OUD and heavy fentanyl use, who were successfully started on a BUP-XR depot following 48-hour induction with BUP-TD. Both patients engaged in fentanyl within 24 h of admission but quickly reached therapeutic doses of buprenorphine with minimal withdrawal symptoms. The addition of BUP-XR initiation following rapid induction with the IPPAS method can simplify treatment initiation while carrying the benefits of BUP-XR, which include higher patient satisfaction, fewer missed doses, fewer healthcare visits, and lower risk of relapse and therefore death by overdose [10, 17, 18]. Furthermore, the motivation of patients to stop or reduce their use of unregulated opioids can ebb and flow, so a shortened induction can optimize moments of enhanced motivation to transition them to life-saving OAT. This is particularly critical in the fentanyl era given the alarmingly high risks of relapse and overdose.

As with Case 1 receiving PRN SL BUP/NX after his BUP-XR injection, it should be noted that supplementation with BUP/NX during the early months of BUP-XR may be needed to manage breakthrough symptoms of OUD due to lower buprenorphine serum levels in the initial treatment period. Case 2 was immediately discharged after her BUP-XR injection, so she did not receive additional doses of BUP/NX in hospital.

Our approach may be especially well-suited to the outpatient setting given its ease of use compared with other low-dose induction approaches. With buprenorphine being an effective, yet underutilized agent for the treatment of chronic pain, it should also be explored how this strategy can be adapted to this patient population. A disadvantage of the subcutaneous route of administration is the potentially painful injection, which may further be aggravated by needle anxiety, and so both patients indicated a preference for BUP-XR and provided informed consent prior to administration.

An inherent limitation of the case series is its small sample size (*n* = 2), limiting generalizability. Therefore, further research is needed to explore the safety and efficacy of this approach in larger patient populations, different settings, and different patient demographics; we plan to conduct a clinical trial in the near future. A potential safety concern is the large number of TD patches used, which may lead to adverse events if they are forgotten to be removed or to diversion and misuse. However, this can be overcome by nursing and patient education.

BUP-XR in Canada is covered by the publicly funded medication insurance programs of all provinces and territories; while in the United States, it may be difficult to utilize BUP-XR in hospital settings due to regulatory and insurance barriers, and its high cost. Furthermore, in both the United States and Canada, BUP-TD is indicated for pain management but not for OUD treatment, making it challenging to secure medical insurance coverage for this indication and incur costs to the patient. Our hospital’s pharmacy has supported our buprenorphine induction protocols by offering BUP-TD to patients undergoing this induction approach at no cost. While we recognize there are barriers to the use of this induction strategy for some jurisdictions, this case series demonstrates a proof of concept for future studies, which could potentially lead to the eventual removal of legal, regulatory, and market barriers for the benefit of patients with OUD.

Future research may focus on further optimization of the induction protocol. The usage of higher-dosage TD formulations (TRANSTEC 35, 52.5, and 70 µg/h) would reduce the number of patches, though this is currently unavailable in North America. In this study, a test dose of SL BUP/NX was given to determine readiness to receive the BUP-XR injection and thereby reduce the risk of precipitated withdrawal; future studies should explore the feasibility of transitioning directly to BUP-XR from BUP-TD.

## Data Availability

Data will be made available on reasonable request.

## References

[1] Orman JS, Keating GM. Buprenorphine naloxone. Drugs. 2009;69(5):577–607. 19368419 10.2165/00003495-200969050-00006

[2] Whelan P, Remski K. Buprenorphine vs methadone treatment: a review of evidence in both developed and developing worlds. J Neurosci Rural Pract. 2012;3(1):45–50. 22346191 10.4103/0976-3147.91934 PMC3271614

[3] De Aquino JP, Parida S, Sofuoglu M. The Pharmacology of Buprenorphine Microinduction for Opioid Use Disorder. Clin Drug Investig. 2021;41(5):425. 33818748 10.1007/s40261-021-01032-7 PMC8020374

[4] Teruya C, Schwartz RP, Mitchell SG, Hasson AL, Thomas C, Buoncristiani SH, et al. Patient perspectives on buprenorphine/naloxone: a qualitative study of retention during the starting treatment with agonist replacement therapies (START) study. J Psychoact Drugs. 2014;46(5):412–26. 10.1080/02791072.2014.921743 PMC4220245 25364994

[5] Hämmig R, Kemter A, Strasser J, von Bardeleben U, Gugger B, Walter M, et al. Use of microdoses for induction of buprenorphine treatment with overlapping full opioid agonist use: the Bernese method. Subst Abuse Rehabil. 2016;7:99–105. 27499655 10.2147/SAR.S109919 PMC4959756

[6] Wong JSH, Nikoo M, Westenberg JN, Suen JG, Wong JYC, Krausz RM, et al. Comparing rapid micro-induction and standard induction of buprenorphine/naloxone for treatment of opioid use disorder: protocol for an open-label, parallel-group, superiority, randomized controlled trial. Addict Sci Clin Pract. 2021;16(1):1–10. 33579359 10.1186/s13722-021-00220-2 PMC7881636

[7] Azar P, Wong JSH, Jassemi S, Moore E, Vo DX, Nikoo M, et al. A Case Report: Rapid Micro-induction of Buprenorphine/Naloxone to administer Buprenorphine extended-release in an adolescent with severe opioid use disorder. Am J Addict. 2020;29(6):531–5. 32346944 10.1111/ajad.13050

[8] Baumgartner K, Salmo E, Liss D, Devgun J, Mullins M, Galati B, et al. Transdermal buprenorphine for in-hospital transition from full agonist opioids to sublingual buprenorphine: a retrospective observational cohort study. Clin Toxicol (Phila). 2022;60(6):688–93. 35048759 10.1080/15563650.2022.2028802

[9] Hess M, Boesch L, Leisinger R, Stohler R. Transdermal Buprenorphine to switch patients from higher dose methadone to buprenorphine without severe withdrawal symptoms. Am J Addict. 2011;20(5):480–1. 21838850 10.1111/j.1521-0391.2011.00159.x

[10] Azar P, Wong JSH, Mathew N, Vogel M, Perrone J et al. 48-hour induction of Transdermal Buprenorphine to Sublingual Buprenorphine/Naloxone: the IPPAS Method. J Addict Med. 2022. 10.1097/ADM.0000000000001072 36149002

[11] Klaire S, Zivanovic R, Barbic SP, Sandhu R, Mathew N, Azar P. Rapid micro-induction of buprenorphine/naloxone for opioid use disorder in an inpatient setting: a case series. Am J Addict. 2019;28(4):262–5. 30901127 10.1111/ajad.12869

[12] Marsden J, Kelleher M, Gilvarry E, et al. Superiority and cost-effectiveness of monthly extended-release buprenorphine versus daily standard of care medication: a pragmatic, parallel-group, open-label, multicentre, randomised, controlled, phase 3 trial. eClinicalMedicine. 2023;66. 10.1016/j.eclinm.2023.102311. 10.1016/j.eclinm.2023.102311 PMC10692661 38045803

[13] Priestley T, et al. Converting from Transdermal to Buccal formulations of Buprenorphine: a pharmacokinetic Meta-model Simulation in healthy volunteers. Pain Med. 2018;19:1988–96. 29036723 10.1093/pm/pnx235

[14] Jones AK, Ngaimisi E, Gopalakrishnan M, Young MA, Laffont CM. Population Pharmacokinetics of a monthly Buprenorphine Depot Injection for the treatment of opioid use disorder: a combined analysis of phase II and phase III trials. Clin Pharmacokinet. 2021;60:527–40. 33135125 10.1007/s40262-020-00957-0 PMC8016750

[15] RxODE: Facilities for Simulating from ODE-Based Models. R package version 1.1.5. 2022. https://cran.r-project.org/web/packages/RxODE/index.html.

[16] Wesson DR, Ling W. The clinical opiate Withdrawal Scale (COWS). J Psychoact Drugs. 2003;35(2):253–9. 10.1080/02791072.2003.10400007 12924748

[17] Rosenthal RN, Lofwall MR, Kim S, et al. Effect of Buprenorphine implants on Illicit Opioid Use among abstinent adults with opioid dependence treated with Sublingual Buprenorphine: a Randomized Clinical Trial. JAMA. 2016;316(3):282–90. 27434441 10.1001/jama.2016.9382

[18] Lintzeris N, Dunlop AJ, Haber PS, Lubman DI, Graham R, Hutchinson S, Tiberg F. Patient-reported outcomes of treatment of opioid dependence with weekly and monthly subcutaneous depot vs daily sublingual buprenorphine: a randomized clinical trial. JAMA Netw Open. 2021;4(5):e219041–219041. 33970256 10.1001/jamanetworkopen.2021.9041 PMC8111483

